# Flipping Patients and Frames: The Patient in Relational Medicine

**DOI:** 10.5041/RMMJ.10310

**Published:** 2017-07-31

**Authors:** Henri Zukier

**Affiliations:** The Hebrew University of Jerusalem, Jerusalem, Israel

**Keywords:** Agency, meaning, relational, self-image, time focus

## Abstract

Medicine has evolved in two opposite directions. Evidence-based medicine focuses more on laboratory and computer data than on the patient. Yet experimental data also provide growing evidence for the importance of the patient’s social-psychological “demand” side of medicine, to complement the doctor’s bio-cognitive “supply” side. The patient’s mindset has major diagnostic and therapeutic effects. The patient’s experience is shaped by perceptions of four dimensions: meaning, agency, self-image, and temporal focus. The patient’s perceptions are linked in part to the therapeutic context, through the interaction between doctor and patient. In that proximal setting, the dimensions can be reshaped, for better and worse. These dynamics point to the inherently interactional nature of medicine and to the significant role of medical social sciences in the therapeutic context.

## INTRODUCTION

In a 2016 essay on the changed nature of their work, two prominent physicians reflected on the modern practice of medicine. Most of their work and medical attention (estimated to be well over 50%) focused on the computer, the chart, the phone, and other staff, away from the lives, bodies, and identities of their patients.^1^ This “flipped patient” approach,^2^ the authors noted, introduced serious limitations. The approach led to framing biases and created a “dysjunction” between the physician and patients, their mind, their personhood, and embodied identity.

The physician’s distancing from the patient does not reflect individual preferences. It results from the objective, structural tensions confronting every physician, which are inherent in evidence-based medicine and the biophysical model of disease. Vast scientific advances generate massive laboratory results and Big Data, yielding complex cognitive puzzles to be cracked by the computer and algorithms. In addition, every individual case is limited in time and resources. As a result, the focus of medicine has become increasingly narrow, concentrating on “repair of health, not sustenance of the soul.”^3^^(p128)^ Paradoxically, the reduction in patient interaction occurs at the very same time that twenty-first-century medicine also newly reveals the important diagnostic and therapeutic roles of the physician–patient interaction, in a way that was not evident before.

Medical thinkers have underscored the limits of the scientific focus in medicine. Groopman, then the Chief of Experimental Medicine at Beth Israel Deaconness Medical Center, reflected on his earlier medical training. In a book chapter entitled “Unprepared,” he described how helpless he felt in dealing with a patient because his education had not prepared him for matters of the heart: “I mistook information for insight. While I was well prepared for the science, I was pitifully unprepared for the soul.” Groopman’s education reflected the general approach: “Although lip service was paid to the patient’s emotional state, it was largely ignored.” Young doctors did not discuss “the psyche and the soul” but saw their future “in hard science.”^4^ Gawande echoes these concerns: “What worried us was knowledge,” and “it did not take [me] long to realize how unready I was to help them [his patients].”^3^^(p3)^

As the distance between physician and patient has grown, so has the empirical evidence for the inherently relational nature of medicine, for diagnostic and therapeutic purposes. The interaction between doctor and patient, like the interaction between body and mind, is increasingly recognized as an inextricable process and as a central component of therapeutic intervention. The patient’s mindset moved from the sidelines, in need of supportive care, to become an integral part of the medical process. The approach is not a throwback to an earlier ethos of caring “bedside” manners by a nurturing house doctor; it is a significant scientific advance that elucidates the instrumental role of psycho-social variables. The mind has moved from being seen as noise and error in the system, into being considered an essential component of the medical process with significant impact and importance.

The role of intuitive cognitive and psycho-social variables in intervention has already produced paradigm shifts in other fields, including economics. The science of decision-making and behavior in many disciplines has been transformed by the seminal work of two Israeli psychologists, Kahneman and Tversky.^5^,^6^ Kahneman and Tversky challenged the common core idea that people are inherently logical and tend to follow normative principles of decision and behavior. In the traditional view, observed departures from rationality were typically attributed to the intrusion of extraneous factors, including emotions. Kahneman and Tversky argued instead that the prescriptive model of human behavior was insufficient. A complementary approach was needed to provide a descriptive understanding of what people were actually doing. People’s choices and behaviors were also guided by so-called heuristic processing, involving the use of intuitive, non-normative mental rules for decision-making. The heuristics were cognitive devices that were often useful but also led to systematic errors. In contrast to prior conceptions of departures from rationality, the heuristics work showed that the errors arose from cognitive processes, not affective corruption, and that they were systematic and predictable. Once the cognitive dynamics were recognized, they could be harnessed to direct change.

The impact of the psychological work was transformative in economics, leading to the rise of “behavioral economics.” Tversky and Kahneman’s work, and related advances, was recognized by the 2002 Nobel Prize in Economics. Economists noted that since the intuitive mental rules reliably produced predictable outcomes, they could become valuable assets for designing public policies, and provide effective leverage for change. Thaler and Sunstein, prominent economist and legal scholar, respectively, described a “choice architecture” approach that engages the intuitive dynamics for systematic change. Many nations already incorporate the insights into the institutional design of policy. Sunstein noted that psychological research had helped inform initiatives in finance, highway safety, consumer protection, energy, climate change, obesity, poverty, crime, and many other areas. The law professor and former government official urged the creation of a national council of psychological advisers, on the model of the councils of economic advisers in place in many nations.^7^,^8^ In a related public policy area, Fischhoff, another prominent heuristics researcher, has described how the US Food and Drug Administration (FDA), which regulates products that account for 20% of US consumer spending, also seeks to integrate the heuristics insights. The FDA realized that its success depended on a proper understanding of human behavior, requiring insights into both products and consumers. As a consequence, the FDA has increasingly made psychology integral to its processes.^9^

Medical thinkers also considered the role of mind in medicine. Their work focused primarily on doctors’ cognitive mindsets. They described potential pitfalls in medical decision-making for doctors and patients, and ways to optimize decisions.^3^,^4^,^10^,^11^ These works also consider, for example, how to navigate conflicting medical advice, or conflicts of treatment preferences between doctors and patients. Some work focused in particular on the communication process between doctors and patients; others emphasized the need for a doctor’s mindfulness to cultivate an open mind and cognitive flexibility.^12^,^13^

This paper considers several additional psycho-social dimensions, centered on the patient’s subjective experience and on the relation with the physician. Work in psychology suggests that the features of the patient’s subjective experience also play a significant role in medical care and in therapeutic outcomes. The paper argues for enlarging consideration of the relational context of medical practice, and of the effects of psycho-social variables on biomedical developments. Attention to the patient’s subjective experience complements the focus on the doctors’ cognitive mindset and decision-making dynamics. This perspective combines a focus on the supply side of medical practice (how doctors think, feel, diagnose, and treat) with a focus on the patient’s human “demand side” of the relationship. Both sides are an integral part of the inherently relational nature of medicine.^14^

The paper explores four core variables that arise in part from the doctor–patient relationship: meaning, purpose; agency and personal control; autonomy and self-image; and temporal focus. The research findings have often developed within psychology,^15^–^17^ with documented beneficial effects but with limited resonance in medicine. Illness is a frightening and disorienting experience. Like other traumas, it shatters people’s fundamental assumptions about the world and themselves.^18^ Finding meaning relieves the intolerable sense of randomness often accompanying trauma, and is often beneficial.^19^ Agency and personal control are defined as a person’s sense to be able to influence outcomes. A sense of self-efficacy, even if limited in scope, provides motivation to act and to persevere in the face of difficulties.^14^,^20^ A positive sense of self, including the experience that one’s voice and preferences are recognized in the medical interaction, also has beneficial effects.^21^ Finally, disease tends drastically to narrow the patient’s temporal focus on the present circumstances, to the detriment of other considerations. Enlarging the temporal focus redirects attention to possible different futures, and can provide motivation and determination to reach for them.^22^

The reactions and resources in coping with illness are typically conceptualized as a form of response to trauma. The clinical analyses usually include few detailed accounts by victims of trauma. There is, however, a field of trauma with ample reflections of individuals, distilling the core of their experience from close up. The literature by and on Holocaust survivors provides insightful accounts with striking parallels and convergence with the empirical findings of traditional illness and trauma research. The extremity of the Holocaust experience does not entail a clinical exceptionalism in processes of resilience. Rather, the extremity serves as a magnifying glass of sorts, bringing the processes in sharp relief. Such accounts complement the clinical research picture and underscore the convergence in processes. Indeed, there has been a lively two-way traffic between the Holocaust and psychological literatures: psychiatrists have developed entire theories of resilience and coping based on their own and others’ Holocaust experiences,^23^,^24^ and investigators of resilience not infrequently point to the Holocaust for parallels.^25^–^27^

## RELATIONAL MEDICINE: PSYCHO-SOCIAL VARIABLES FROM EXTRANEOUS LIABILITY TO ESSENTIAL LEVERAGE

The role of psycho-social variables in the evolution of illness, and the relational nature of medicine, comes into relief in the dynamics of the doctor–patient relationship. Psychoanalysis and related therapeutic interventions in mental health early recognized the centrality of the clinical relationship in the process, as a potential liability, to be turned into a powerful leverage. According to Freud, the charged relationship, in which the patient predictably develops negative and erotic feelings to the analyst, was one of the chief expressions of the patient’s resistance to the treatment and the main obstacle to therapeutic progression. The analytic process was “the battlefield of transference.” The analyst strove to defeat the neurotic impulses by entering an alliance with the patient. In this way transference turned from “the most powerful resistance to treatment” into its “main instrument.”^28^ The relational roadblock had been transformed into the change mechanism itself. Freud’s work on affective dynamics shared fundamental features with the recent work on cognitive heuristics: both adopted a descriptive approach that did not treat deviations from norm as deviance; both revealed the systematic patterns of the anomalies, their predictability and meaning; and both then set out to harness these findings to promote change.

The idea of an instrumental therapeutic alliance between physician and patient developed across the mental health fields, even in approaches (such as cognitive-behavioral therapies) far removed from psychoanalysis. Previously, therapeutic effects were attributed to various technical modalities, with the quality of the relational bond between patient and therapist treated as an added value. Recent research and meta-analyses indicate that the quality of the relationship itself is in fact an integral component of the process, and a robust predictor of outcomes, across a wide range of diagnoses and treatments.^29^,^30^

The growing recognition of the importance of the psycho-social context has also become evident in the evolving perspective on the placebo effect. The placebo has a long and distinguished history as a negative, morally reprehensible sham intervention, associated with deception. In Chaucer’s fourteenth-century *Canterbury Tales*, Placebo is, in one tale, a dishonorable, flattering character who causes significant harm. In another tale, flatterers are called the devil’s chaplains, always singing placebo.^31^,^32^ Donizetti’s nineteenth-century opera *L’Elisir d’Amore* features the traveling quack doctor Dulcamara, a “doctor sans pareil,” who takes advantage of the credulity of the villagers. His potion, he promises, will cure old age**,** banish wrinkles, move the paralytics, cure the apoplectics, asthmatics, diabetics, and most other maladies.

In medicine, like in literature and opera, the placebo was long considered a harmless substance (sugar pills) or intervention that aimed to appease or “please” (“placebo” in Latin) patients by fostering an illusion of treatment when no intervention was available or called for. A focus on the bio-physical features of disease made the negative placebo conception self-evident. Since the placebo was an inert substance or treatment, it could not possibly have a therapeutic effect, by definition.^33^ Yet growing empirical evidence suggested that placebo effects occurred in various contexts. This realization focused medical attention on the psycho-social dimensions of the clinical situation, and on the larger affective challenges inherent in medicine, for physician and patient. There has been a re-evaluation of the placebo effect and a recognition of its clinical role.^34^

The importance of the psycho-social context in clinical care has been shown, for instance, in the so-called “open–hidden” paradigm. In the “open” condition, like in standard care, the clinician openly administers the drug or treatment and interacts with the patient. In the “hidden” condition, the treatment is administered without the patient’s awareness, and in the absence of the usual clinical social context. Studies have shown that the hidden administration of five common painkillers is significantly less effective than their open administration, and similar effects have been shown for other conditions.^35^,^36^ The results suggest that the overall outcome of a therapy involves a combination of effects: a specific physiological action, and a psycho-social context effect. The medical context influences the outcome primarily through the patient’s expectations and the physician–patient communications.^37^,^38^ The therapeutic potential of placebo effects has led to calls to “harness” it and to incorporate it in clinical care.^39^

## INTERPRETERS OF MALADIES

A fundamental aspect of human experience involves a fear of randomness, unpredictability, and loss of control. People are particularly apprehensive about personal or public hazards (to their health or security, for instance) that are ill-defined, undetectable, or that can strike at any moment. Threats of disease or terrorism often evoke distress (as the term “terror” suggests) out of proportion to the objective risks and consequences.^40^ This tendency reflects a fundamental psychological need to find order in chaos. To ensure a coherent (and predictable) environment, people will generate intuitive explanations of events that privilege perceived cause–effect relations. The accounts can erroneously identify patterns in random occurrences, or attribute events to a wrong causality. Psychologists have described the pervasive human tendency to interpret sequences of physical events or social interactions in cause–effect terms. Six-month-old infants already perceive events in terms of cause–effect relationships, as suggested by their longer looking time at events that violate their expectations, compared to confirmatory events. The “illusion of causality” that arises in infancy remains a powerful and prevalent influence in adult thinking.^5^,^41^–^43^

Stories, or narratives, are one intuitive way to connect the dots, and generate causality. Narratives serve children and parents alike to make sense of the world and construct meaning. Neuroscientists have demonstrated the human narrative tendency in split-brain patients. In such conditions, the left hemisphere and language center do not have access to information that is presented to the right brain. In a series of studies, researchers showed, for instance, a scary fire video to a patient’s right hemisphere, triggering an emotional response of fear and anguish. When asked to explain her reaction, the patient’s left hemisphere speech center (unaware of the scary scene) improvised an explanation, saying that the experimenter was somehow scary. The experimenters also flashed the picture of a pin-up girl to the right hemisphere of a patient, who started laughing. When asked to explain her emotion, she said there was a funny machine in the room. The researchers called the left-hemisphere process that created *post hoc* confabulations for ill-understood experiences *the interpreter*.^44^ We are all “interpreters of maladies”^45^ in everyday life, constructing our lives and ourselves as coherent narratives. The narratives impose order on disorder, as if order were the expectable baseline in life.

Medical anthropologists distinguish between disease and illness. Disease is the objective biomedical condition that the physician fits into a technical nomenclature and taxonomy to create a diagnostic entity. Illness refers to the subjective, lived experience of symptoms and suffering, to the patient’s understandings and expectancies, and to the socio-cultural meanings associated with the disease. The uncertainty, injustice, and suffering of illness are given coherence in “illness narratives” developed by patients and society.^46^ The “illness narratives” embody the psycho-social dimension that physicians have to contend with.

The Book of Job opens, in its very first verse, with God’s categorical affirmation that Job was blameless and upright. The chapter then proceeds to describe how Job, in a breathtaking sequence, loses his material possessions, his children, and then is struck with body-wide loathsome sores. Upon hearing of his great suffering, Job’s friends immediately converge to “console and comfort him.” Each in his own way explains Job’s suffering in terms of a “moral causal” model for misfortune. Surely, they suggest, Job’s suffering was connected to some known or unknown transgressions; in some way, the sufferer *must be* at fault. After 35 painful chapters of Job’s friends blaming the victim, God Himself can take it no more. He first appears to Job to proclaim again Job’s righteousness, without, however, offering an explanation for Job’s immense sufferings. God merely reaffirms the inherent limits of human understanding. He then turns to Job’s friends, the unknowing “interpreters.” God rebukes them for offering the standard spurious moralistic “consolations” for illness.

Job’s friends were hardly alone in evoking a moral explanatory model for misfortune. Anthropologists have identified several kinds of explanations of suffering that are prevalent, in local idiom, in most societies: interpersonal, moral, and biomedical modes of causal explanation. The moral mode of explanation, in particular, sees suffering as linked to the victim’s own actions or intentions, sinful thoughts, and acts of commission or omission.^47^

The four aspects of the subjective experience of illness considered in the paper are core elements in patients’ explanatory models. The elements are shaped, and can be modified, by the interactional context of the patient’s relation with the physician, and often have a significant effect on the course of illness. Meta-analytic studies have shown that psychological well-being has a protective effect and can enhance long-term survival in both healthy and diseased populations.^48^

## REPRESENTING THE CONTEXT: MEANING AND PURPOSE

Concentration camp prisoners noted the protective effects of a “larger” meaning for prisoners. Frankl, the Austrian psychiatrist, reports how he was struggling “to find the *reason* for … [his] sufferings,” recalling Nietzsche’s words that “He who has a *why* to live for, can bear with almost any *how*.” What was needed for survival was “a fundamental change in our attitude toward life.”^23^^(pp60,97,98)^ Frankl drew on his experiences in the Nazi camps to develop his existential therapy, logotherapy, focused on people’s need for meaning. Frankl recounts how he warded off two would-be suicides in the camp by turning their expectations to people and purposes. Both prisoners believed they had nothing more to expect from life. Frankl insisted, instead, on what life still expected of them. For one, it was the love of a child waiting for him in a foreign country; for the other, completing a series of unfinished scientific books.^23^^(pp100–1)^

Camp prisoners all observed that ideologues fared better than ordinary inmates. Primo Levi, echoing Jean Améry, both agnostics, noted that “Not only during the crucial moments of the selection or the aerial bombings, but also in the grind of everyday life, the believers lived better … it was completely unimportant what their religion or political faith might be. Catholic … Zionists … Marxists and Jehovah’s Witnesses … Their universe was vaster than ours … more comprehensible. They had a key and a point of leverage … so that there might be a sense to sacrificing themselves … sorrow … was decipherable and therefore did not overflow into despair.”^49^^(p118)^ The Nazis also understood the corrosive effects of lack of meaning. A hallmark of the ghettos and camps was the utter arbitrariness of anti-Jewish policies, even to the point of conflict with other German war priorities.^50^,^51^ Primo Levi captured in one brief exchange the Germans’ effort to drain all meaning from the prisoners’ experience, and thus to destroy their humanity and resilience even before their physical annihilation. “*Warum?*” (Why?), Levi asked a brutal camp guard, who answered: “*Hier ist kein warum*” (there is no why here).^52^^(p35)^ For many prisoners, a moral mission “to bear witness” to the meaningless horror itself became a “why” in their efforts for survival.

A similar turn toward a larger purpose and meaning also is essential in everyday life. It enables people to transcend the immediate context in favor of more distant and important objectives. Decades of studies by Mischel on what became known as the “marshmallow test” showed how pre-schoolers’ differences in representing situations made it “either impossibly difficult or remarkably easy” to resist short-term gratification for a larger long-term goal, and that the differences carried forward into adult life and professional and personal success.^53^ Clinical evidence also points to the connection between a sense of purpose and ego-resilience. In two longitudinal cohort studies of community-dwelling elderly persons, a higher level of purpose in life was associated with a substantially reduced risk (40%) of all-cause mortality, during a follow-up period of up to 5 years. The association of purpose with mortality did not vary by age, gender, or education, and persisted after control for several covariates. The authors concluded that the tendency to derive meaning from life’s experiences and possess a sense of goal-directedness contributed to longevity.^54^–^57^ Similarly, a prospective study of religiousness and recovery from heart surgery found that stronger religious beliefs were associated prospectively with fewer surgical complications and shorter hospital stays. More broadly, religious involvement has repeatedly been associated with lower risk of all-cause mortality in large populations.^58^,^59^ A reconceptualization of the illness experience can help infuse it with new meaning. Survivors often attribute positive life changes to the experience of disease, leading them to reorder their priorities, values, and relationships. The process has been termed post-traumatic growth. A number of studies demonstrated a pattern of post-traumatic growth in cancer patients and others.^60^–^62^

## REPRESENTING THE SELF: PERCEPTIONS OF PERSONAL CONTROL AND AGENCY

Soon after his arrival in Auschwitz, Primo Levi received a lesson in survival from an experienced older inmate. The prisoner urged Levi not to neglect his care of self, and to remain meticulous with washing and cleanliness practices. At first, Levi was scornful about the patently absurd advice: why wash in filthy water, and in pervasively squalid conditions that would instantly nullify any fleeting hygiene? Better preserve one’s very limited energies for life. But soon Levi realized that it was not a hygienic recommendation at all, but rather an essential means for preserving one’s self-worth and moral survival. The Nazis engaged in systematic “excremental assaults” in the camps, forcing prisoners to live in squalid conditions. These measures were designed to hollow out the inmates psychologically and morally, and to reduce them to beasts, in the eyes of their captors and in their own. Washing was necessary for “dignity and propriety,” and “not to begin to die.”^63^^(pp40–1)^

The seminal work on personal control and health was conducted by Langer and Rodin, and Bandura expanded on the role of self-efficacy across a large variety of domains.^14^,^20^,^25^,^64^ Langer and Rodin gave a group of elderly nursing home residents the opportunity to assume “more responsibility” and make more choices in their daily lives. The residents could make decisions about the arrangement of their room furniture, about people and activities in their daily schedules, and were offered the responsibility of caring for a plant. In the control group, the staff assured the residents that the staff would care for them and try to make them happy. Behavioral measures and nurses’ ratings showed significant improvement across a variety of dimensions for the residents offered more responsibility over their lives. The beneficial effects could still be observed 18 months later. Most strikingly, the “responsibility” group had a significantly lower mortality rate during that period. Real-life changeovers of nursing homes, aiming to provide residents not only comfort, but also responsibilities, autonomy, and commitments transcending their daily routines, also showed dramatic effects.^3^ Dweck showed, more generally, how people’s views of themselves (their mindsets), their beliefs about their ability to influence events, to learn and to grow, affect the way they actually lead their lives and who they become. These qualities are not naturally given, but must be cultivated, at first by parents, teachers, and other figures in a person’s life.^65^ They can also be nurtured in the therapeutic interaction.

In studies, a sense of self-efficacy has been found to be significantly correlated to a patient’s adjustment, coping, and resilience, and to the actual course of disease and treatment. In children, a sense of personal control was associated with better asthma control. In adults, longitudinal studies of heart disease patients indicated that a perception of control was associated with better adjustment to the disease, and similar links were also evident in breast cancer patients. A sense of control among patients with coronary artery disease after angioplasty also predicted a reduced likelihood of sustaining another cardiac event over 4 years.^66^–^69^ Patient expectancies of efficacy were also predictors of survival in people with chronic obstructive pulmonary disease, and predicted recovery of function and reduction in symptomatology after total knee replacement surgery.^70^,^71^

## EXPERIENCING THE SELF: SELF-IMAGE AND AUTONOMY

Job had the inner strength to reject his friends’ moralizing intimations of guilt and shame. Many patients and other victims do not. Instead, they initiate, or internalize, social stigmas attached to their condition.

In a chapter entitled “Shame,” Primo Levi recounted how shame persisted among his fellow Auschwitz prisoners, though on a rational plane they had nothing to be ashamed of.

... That many (and myself) experienced ‘shame’, that is, a feeling of guilt during the imprisonment and afterwards is an ascertained fact … It may seem absurd, but it does exist.^49^^(pp54,58,62)^

Guilt, shame, and misplaced causal attributions are also common among rape victims, medical patients, and victims of other uncontrollable catastrophes.^72^ In a study of breast cancer, 95% of the patients developed a theory about their disease, with nearly half blaming themselves for it, and over half feeling they had personally some or a lot of control over the course of the cancer.^71^ In another study, a majority of people who had suddenly lost a child or a spouse in a car accident 4 to 7 years earlier still had the recurrent thought that they could have prevented the accident if only they had done something differently.^72^,^73^ In 1976, a group of American children was kidnapped, and released physically unharmed 27 hours later. Five years after the kidnapping, most of the children had “framed” the trauma and linked it to some of their own guilty thoughts or behaviors, months or years prior to the kidnapping. A 5-year-old girl blamed herself for having stepped in “a bad luck square.” The imaginary transgressions and “omens” transformed the children’s inexplicable ordeal into something intelligible and controllable that could potentially be avoided with proper precautions.^74^

Groopman described the story of a 29-year-old New York Orthodox Jewish woman who long delayed seeking attention for a very large cancerous breast mass, and then resisted treatment. She saw her cancer “as a punishment from God.”^4^ Patients can experience their conditions as bodily signs, stigmas that exposed a reprehensible moral status for all to see. The stigma is not a personal attribute, but a feature of relationships.^75^,^76^ Its force derives from social attitudes to behaviors and qualities that deviate from the norm. Eventually, stigma can destroy the self-respect and the sense of autonomy of the victim, and drive them to self-destruction.

The antidote, in a medical setting, is a relationship that preserves the patient’s self-respect and dignity, by recognizing the patient’s voice and preferences, and engaging in shared decision-making. Decisions about medical treatments typically entail uncertainty and hard choices about outcomes and trade-offs. They may also lead to clashes between a patient’s preferences (autonomy) and what the doctor believes is in the patient’s best interests (beneficence). The conflicts can be most acute in end-of-life decisions. Ethically and empirically, the traditional paternalistic approach to such medical decisions undermines the patient’s experience.^3^,^11^

## REPRESENTING THE FUTURE: TEMPORAL FOCUS

Temporal focus also has a significant influence on motivation and action, and affects people’s determination and efforts to achieve their goals. People’s commitments are a function of the desirability of the goals, of the goals’ temporal distance, and of people’s perceived ability to influence the outcome.^77^,^78^ Every chronicler of the Holocaust has described the desperate figures of the so-called Muselmänner, hollowed out of any view of a possible future, stripped of all motivation and ability to struggle for survival. Frankl, for instance, noted that the source of inner strength for camp prisoners was a future goal to which they could look forward. The prisoners who had lost faith in the future were doomed.^23^ They were easily spotted by the other prisoners: the walking dead, shadows shuffling through the camps and through life for just a little longer.

Patients also adopt temporal perspectives that can incline them to hope and the future, or hopelessness and helplessness.^4^,^15^,^79^ Initial research indicated that victimization could induce a state of “learned helplessness,” characterized by emotional numbing and maladaptive passivity. The attitude was generated by a belief in the futility of trying to cope, and by the expectation that the condition would be enduring (narrow temporal focus) due to action–outcome independence (a loss of agency and autonomy). The model was expanded to a general view of hopeless depression, that combined the victim’s negative ideations about the situation and about the self. The conviction of “no way out” is a joint function of a perception of loss of agency and a bias to a time perspective anchored in the present.

Conversely, positive expectancies for the future are linked to better health in a variety of conditions. Prospective cohort studies indicate that optimism is associated with better health outcomes in patients with ischemic heart disease, and with a lower risk for cardiovascular morbidity and for all-cause mortality. A strong and consistent association has been shown between dispositional optimism and a nearly 50% lower risk of cardiovascular mortality in elderly men over 15 years of follow-up. After coronary artery bypass surgery, optimism was a significant predictor of surgical outcomes and of a faster rate of physical recovery during hospitalization. In older adults, dispositional optimism was also associated with a decreased risk of cognitive impairment over a 4-year follow-up.^4^,^80^–^82^

A patient’s negative bias toward their current condition, and toward a short-term rather than longer-term temporal focus, arises from two fundamental modes of human thinking, so-called systems 1 and 2, or the “hot” and “cool” systems.^5^,^53^ The hot system involves quick, reflexive, and more automatic responses; the cool system is more reflective, problem-solving, and cognitive. The hot system is focused on the present; the cool system is more future-oriented, promoted by a sense of agency and choice. Pain, for instance, is a “hot” stimulus that can lead to immediate, relief-oriented actions that may not be optimal over the longer term. The quick decisions resemble everyday dilemmas of self-control. The hot system grasps at immediate rewards, real and imagined, and heavily discounts future outcomes, including less favorable results of a medical intervention, or possible adverse effects. Emotional arousal is linked to an impulse for present action, though the reflective perspective understands the possible exaggeration of benefits and the underestimation of risks and costs. This “focusing illusion” on the present seizes on the most salient, vivid, and readily available features of a situation, which are in the present and privilege concrete and near-term action.

The multiple personal and social factors that influence a patient’s ability to respond to illness with resilience or hopelessness clearly interact with each other and can enhance or reduce the likelihood of particular outcomes. The interactions require further research, as do the generalizations, with a need to outline qualifications and limiting conditions. Yet it remains clear that doctors can help.

## THE DOCTOR’S INTERPERSONAL ROLE: FLIPPING FRAMES OF AGENCY, SELF, AND TIME

During the Holocaust, victims often drew resilience from small acts of kindness by ordinary people. Victor Klemperer, the 60-year-old literary scholar and diarist from Dresden (spared deportation, but not persecution, because of his Aryan wife), recorded the kindnesses, small and large, from strangers and friends, that sustained the couple throughout the war. The gestures, Klemperer noted, may not help him in the end. But they provided comfort and consolation.^83^,^84^ As did the demeanor of a memorable literary doctor, George Bernard Shaw’s B. B., who was so “cheering, reassuring, healing by the mere incompatibility of disease or anxiety with his welcome presence. Even broken bones, it is said, have been known to unite at the sound of his voice.”^85^

A demonstration of the ease and power of reversing, or flipping, decision frames and attitudes, for patients and doctors alike, was provided in a classic article by Tversky and colleagues, “On the elicitation of preferences for alternative therapies.” In the study, groups of patients and Harvard physicians were asked to choose between two treatments for lung cancer: surgery or radiation. They were all given data about the outcomes of the two treatments, which indicated that surgery yielded a clear 5-year advantage for life expectancy, but posed a slightly larger immediate risk than radiation. Half the participants received the data framed in terms of survival rates; the other half received the same data framed as mortality rates. The differential framing of the equivalent choices had a major effect. Both patients and doctors favored surgery over radiation far more often when the data were presented as survival rates instead of mortality rates. As Kahneman noted, the hot system is “rarely indifferent to emotional words” such as mortality or survival. It also gives much more weight to loss aversion, and to its prevention, than to an equivalent gain. Further data indicated that public-health professionals were equally susceptible to the framing of alternative public policies as “lives saved” or as “lives lost” scenarios.^5^,^86^

Another classic example also epitomized how patients’ focus can be shifted away from the hot present, to a cooler, reflective longer-term perspective. In the story of Job, God intervened to cool the “hot” moralistic arguments of Job’s friends, and rebuked them for offering “darkened counsel” and speaking “without understanding.” In a memorable essay, “The median is not the message,” the evolutionary biologist S. J. Gould articulated the limits of knowability about his own catastrophic disease.^87^ At age 40, Gould was diagnosed with abdominal mesothelioma, which, according to statistics, was an incurable cancer with a median mortality of 8 months after discovery. Gould reframed the popular perception of the statistics for disease. Statistical averages, such as median survival rates, he noted, were only abstractions that could not account for the variations (of the disease). Yet variation, rather than the average, was “nature’s only irreducible essence” and “hard reality.” The realization of the right-skewed profile of the distribution of his disease, that is, of the inherent unknowability of the individual complexities of its course, gave him “solace.” Gould lived another 20 years, and died of an unrelated cancer. He also embodied the beneficial role doctors can play in properly shifting patients’ attitudes and actions away from a “hot” anchor of fear and loss that discounts the future, to a more prospective cooler cognition.

## CONCLUSION: TOWARD RELATIONAL INTERVENTIONS IN MEDICAL CARE

Medicine has recently evolved in two opposite directions. On one hand, evidence-based medicine privileges the laboratory and the computer over the patient, both by necessity and choice. On the other hand, the experimental data also provide ever more evidence for the importance of the patient’s psycho-social “demand” side of medicine, in contrast to the doctor’s bio-cognitive “supply” side. The context of the intervention and the patient’s mindset have major diagnostic and therapeutic effects. The patient’s experience is shaped by perceptions of meaning, agency, self-esteem, and the future. At first sight, these would seem personality factors and long-term, large-scale dimensions not easily amenable to change. But there still is considerable room for more focused and manageable interventions. For the patient’s experiences also arise from within the therapeutic context, through the interaction with the doctor. In that proximal setting, the dimensions can be reshaped, for better and worse. A transformation of the patient’s sense of agency or hope, say, need not yield a resolution of the central issue (nor did Primo Levi’s washing in the camps). However, local reaffirmations of self, reorientations of time, or reframings of meaning (as were commonly practiced during the Holocaust) may be powerful and easy interventions critical to the long-term outcomes. They reflect the inherently interactional nature of medicine and emphasize the role of medical social sciences in the therapeutic context.
